# Medical and Physical Intelligence

**Published:** 1801-05

**Authors:** 


### MEDICAL AND PHYSICAL INTELLIGENCE.

Prof. HARLES relates in Hufeland's Journal, (vol. ix.) a remarkable caſe of morbus maculoſus hæmorrhagicus, attended with dropſy; but as the whole hiſtory of it is too long to be inſerted in this Journal, we ſhall content ourſelves in communicating the moſt eſſential reſults the Profeſſor has adjoined to this hiſtory, which are as follow: 1. The morbus maculoſus hæmorrhagicus and the chronical petechiæ, or petechiæ ſine febre, are, both with reſpect to their form and nature, one and the ſame diſeaſe, as appears from the deſcription of the caſes of theſe diſeaſes given by different authors. 2. The chronical petechiæ and the petechial fever, or the malignant petechiæ, eſſentially differ from each other, and only agree ſomewhat in the external appearance of the ſpots. The acute or malignant petechiæ are diſtinguiſhed from the chronical ones by being attended with a fever of the typhus kind, or ſometimes being a high degree of ſynochus and with ſymptoms evidently ſhowing a ftatus nervofus, and by being moftly epidemic. Both difeafes however, have a great fimilarity in the fpots, though in the chronical petechiæ, vibices, ecchymofes, and other irregular fpots, are frequently remarked, but which feldom occur in the petechial fever. 3. The real fcurvy and the chronical petechiæ are two difeafes effentially different from one another. The fea-fcurvy is attended with a greater debility of the mufcular fibre, want of digeftion, pains in all the mufcles, difficult breathing, fwelling and bleeding of the gums, &c. The fpots are more of a violet, and fometimes of a yellowifh colour, and in its laft ftage hæmorrhages and gangrene of the lungs come on; malignant ulcers are alfo frequently obferved, &c. 4. All thefe difeafes agree in having their origin in a particular morbid ftate of the venous blood in the fmaller cutaneous veffels; and they feem to prove that there probably exift humoral difeafes. This, however, Mr. Harles intends to fhow more extenfively in a publication that is fhortly to appear "In the Apology for Humoral Difeafes," with which we fhall not fail to acquaint our readers more fully as foon as it comes to our hands.

---

Mr. Brefeld has made fome remarks on the ufe of oils, which have fometimes been applied with fuccefs in arthritic complaints. In order to explain their action, he conceives the following idea of the nature of gout. He ftates, that the gout originates in a corruption of the fynovial liquor, which is probably a rancidity of it, as, according to chemical analyfis, the fynovial liquor feems to poffefs the properties of grofs oils, and is confequently expofed to the fame chemical laws. For curing the gout, it is chiefly requifite to correct the corruption of the fynovial liquor, and to prevent its becoming any more rancid; the firft of which is effected by the grofs oils, by involving the acrimony of it, and rendering it thereby more fit for being abforbed by the lymphatic veffels, and removed from the body by the purifying organs. Though this may be obtained, the defect of that liquor replaced, and the increafed action in the folidum vivum diminifhed by the application of grofs oils, yet there are cafes in which corroborant remedies are required for preventing the rancidity of the fynovial liquor. Mr. Brefeld relates a cafe of a young man who was cured of the gout, attended with a cough, by taking feveral times a day a fpoon full of fweet oil, and by frictions of fweet oil and camphor applied between the fhoulders.

---

The fame gentleman recommends wafhing of the belly with cold water, as of great avail in dyfentery. For, fays he, thereby the folid parts are not only ftrengthened, and the increafed tendency of the fluids to corruption greatly counteracted, but it likewife excites motions by which any obftructions are removed, the inflammatory and eryfipelatous ftate of the bowels, and the topical fenfibility of the affected parts, in a great meafure, diminifhed. He found it of good effect in a dyfenteric epidemy, to drink cold water; water; and frequently, when opiates and antimonials were without advantage, cold water, repeatedly drank, diminiſhed the pains in a ſhort time, and rendered the ſtools feculent: When the patients were much debilitated, it was mixed with a little wine.

---

Citizen Lassus relates an intereſting caſe of the deleterious effects of opium. A woman, ſixty years of age, who had been frequently attacked by fits of melancholy, ſwallowed about midnight 36 grains of opium. Five or ſix hours after, ſhe was found to be faſt aſleep, her reſpiration rattling like that of an apoplectic perſon. She recovered, however, for a few moments, ſo as to be able to inform thoſe who attended her, of the quantity of opium ſhe had taken. Ipecacuanha was adminiſtered, but without producing vomiting; after which a few table ſpoons full of vinegar were given to her, which ſhe ſwallowed with much difficulty. It was at this time that Cit. Laſſus ſaw her. She was not ſenſible of what was paſſing around her, and not the leaſt ſign of feeling was left. The reſpiration was attended with noiſe and difficulty, the ſkin moiſt, the pulſe quick, and the pupils of both eyes were conſiderably dilated; the joints were flexible, and every muſcle in a ſtate of relaxation. She died about eleven hours after ſhe had taken the opium.

On diſſecting the body, all the inner ſuperficies of the ſtomach was found inflamed, and likewiſe eroded in ſeveral parts. The inflammation extended itſelf through all the ſmall inteſtines in which large gangrenous ſpots of a greeniſh colour were obſerved. The cavity of the ſtomach contained about ſix table ſpoons full of a turbid liquor of a reddiſh colour, conſiſting of vinegar, ipecacuanha, of the gaſtric liquor, and probably of opium, the laſt being ſo perfectly diſſolved that not the ſmalleſt particle could be traced in it. The ſmall inteſtines were much relaxed, but the cœcum and colon inflated; the reſt of the inteſtines were in a ſound ſtate. There was nothing preternatural found in the brain; and the veins were neither filled with too much blood, nor did the ventricles of the brain contain ſerum; in ſhort, the deleterious effects were confined to the ſtomach and ſmall inteſtines. It appears, therefore, as the author remarks, 1. That opium, when taken in a great quantity, does not cauſe apoplexy, i. e. it produces no congeſtions of the blood in the brain; 2. That the ſooner it is diſſolved in the ſtomach, the ſooner it occaſions, by its irritation, an inflammation which at laſt ends in gangrene; 3. That vegetable acids, which have been ſo much praiſed as antidotes, have no effect at all, unleſs the opium has been taken in ſmall doſes.

---

Cit. Saucerotte communicates, in the ſame work, a very uncommon caſe of a preternatural ſwelling and growth of the bones. A man of middle ſize, in the 35th year of his age, obſerved that all the bones of his body, the teeth excepted, were growing thicker by degrees, but without increaſing in length, and continued advancing till they had reached to double their natural thickneſs. The muſcles and fleſhy parts became relaxed and ſoft, but without feemingly to decreafe in fize; the bones of the fkull had fo much increafed, that the head was of a prodigious appearance, and the eyes protuberated from the thicknefs of the bones of the orbita; the ribs touched one another, and the fternum, clavicula, and the fcapula were uncommonly prominent; the flexibility of the joints was much impeded, and all the motions went on with difficulty. While the bones were thus increafing, the patient became incapable of moving himfelf; he breathed with much trouble and difficulty; the pulfe was hardly to be felt; he fuffered rheumatic pains in every part; and his urine was whitifh with a ftrong fediment. After fix years the bones ceafed to grow, the pains diminifhed, and the refpiration became more free. In the feventh year, however, he died, after having fuffered, about three months, the fame complaints which he experienced during the growth of the bones. It is much to be regretted, that it was not permitted to open the body.

---

Cit. Boucher, in a memoir read before *la Societé d'Emulation at Abbeville*, has given fome obfervations on elm-trees. Thefe trees are frequently attacked by ulcers, from which, at length, a great number of them perifh. *Du Hamel* had already endeavoured to find out the caufe of this difeafe, and afcribed it to a plethora of the fap, an opinion which Cit. Boucher has confirmed by numerous experiments. He propofes at the fame time a remedy, which he fuccefsfully applied to it. He obferved that the local ulcer never attacked the trees on their north-fide, but always on the fouth fide. It acts principally on trees planted in a boggy foil, and along the fide of rivers. Its feat is generally not far from the ground. This ulcer, owing to a fuperabundant fap, differs from a fimilar one, defcribed in *Journal d'Hiftoire Naturelle, No.* 5, 1789, as in this the liquor expofed to the air, foon takes the confiftency of a gum, and has the flavour of fugar.—The remedy confifts in boring every tree attacked by the difeafe, at the ulcer itfelf; and in applying a tube in the hole, occafioned by the borer, penetrating about nine lines in depth. The found trees, which are alfo bored, afford no liquor, whereas thofe that are ulcerated yield it in great abundance, increafing particularly in fine weather, and when the wound is expofed to the fouth. Stormy weather and great winds ftop the effufion. In this manner, he remarks that the ulcers dry and heal in 48 hours. It is probable, that fuch a terebration, tried on other vegetables, and particularly on fruit trees, will produce the fame effects. *Pliny, Columella,* and *Palladius,* have already mentioned this expedient being employed by the ancients, which, however, has not been practifed for many years.—Cit. Boucher concludes by remarking, that this tree is a native of Europe, and has not been lately introduced into France, as is commonly fuppofed; but the writings of the ancients prove, that it exifted there long ago.—The chemical analyfis which he made of the fap, fhewed that it contained a confiderable quantity of acetite of potafh, a little acetite of lime, a certain quantity of vegetable or muco-faccharine matter, and a confiderable proportion of muriate of lime; flight traces of fulphate and muriate of potafh were likewife perceived.

---

A Tranflation of Mr. BAUDELOQUE's Refearches and Reflections on the Cefærean Operation, is in the prefs; to which will be added a Preface, Notes, and an Appendix, with fix engravings, by JOHN HULL, M. D. Manchefter.

---

A popular Medical Work, printed in a full and compact manner, will fpeedily make its appearance from the experienced pen of Dr. ALEXANDER THOMSON. It will contain all the recent difcoveries in the Practice of Phyfic, and a greater variety of matter than in any previous work of the fame defcription.

---

Several Phyficians and Surgeons, in London, have affociated together for the purpofe of writing An Extenfive MEDICAL ENCYCLOPÆDIA; or, An Entire Courfe of the Modern Practice of Phyfic and Surgery; including the Outlines of Anatomy, Phyfiology, Hygiene, Pathology, Nofology, Therapeutics, Midwifery, Pharmacy, Chemiftry, Botany, and Medical Philofophy; with references to the moft approved Authors, and an explanation of technical terms; arranged alphabetically, and illuftrated by numerous engravings. The whole work will be comprehended in two or three quarto volumes, forming a complete MEDICAL LIBRARY; and the names of the different Practitioners engaged in this undertaking are to be in the Profpectus, which will fpeedily appear. We underftand, that it is intended to annex the fignature of each writer to his own articles, after the manner of the French Encyclopædia; and that communications for the Editor may be addreffed to any of the following bookfellers, viz. Meffrs. Longman and Rees, Pater-Nofter Row; Cadell and Davies, Strand; Murray and Highley, Fleet Street; Vernor and Hood, Poultry; or Mr. Cuthell, Middle Row, Holborn.

---

Dr. BATTY will begin his Summer Courfe of Lectures on Midwifery and the Difeafes of Women and Children, on Monday the 8th of June, at half paft ten o'clock in the morning.

---

Dr. REES purpofes to commence his Summer Courfe of Lectures on Midwifery, on the 1ft of June next: For particulars, enquire at his houfe, No. 11, Leicefter Street, Leicefter Square.

---

Mr. CARPUE will commence his Summer Courfe of Anatomical Lectures, on Monday, the 8th of June, at his houfe, No. 44, Leicefter Square. Diffecting will be continued under certain regulations. Particulars may be known by applying as above.

---

### To the EDITORS.

Gentlemen,

As I am convinced it is your wifh, that the interefting publication under your management fhould be an impartial regifter of all ufeful information, I affure myfelf of your readinefs in admitting the following obfervations, occafioned by an account that that appeared in the Medical Journal of February laſt, of an artificial leg, invented by Mr. Potts.

Not having ſeen the inſtrument there recommended, I ſhall not of courſe attempt to call in queſtion its merits. The whole of my intention is, to point out an error Mr. Potts has fallen into, by aſſerting, that till the appearance of his invention, no artificial leg had been produced, in which the perſon uſing it was not obliged to make a ſemicircular motion with it in walking. Now, the following extract from Mr. Mann's deſcription of his patent leg, which I take the liberty of incloſing, will fully prove the miſtake. Mr. P. lies under. "Mr. Mann reſerves it for a perſonal conference with thoſe who may apply, to enter into a detail of particulars, contenting himſelf with aſſerting, that the external appearance of the artificial leg to be ſupplied under the ſanction of this patent, cannot be diſcriminated from the natural, and that the materials are at once light and durable; that it may be applied or taken off in a few moments, without the leaſt pain or trouble to the wearer; that the various circumſtances of ungracefulneſs, rigidity, and inconvenience, ſo viſible in other adſcititious members, are in this obviated; that, as it contains an anatomical and mechanical analogy to the ſeveral articulations to the knee, toes, and foot, and exerciſes all their functions per ſe, merely by being walked on, ſo in ſitting, riding, or ſtanding, it inſtantaneouſly accommodates itſelf to every flexure, extenſion, elevation, and attitude of the natural leg and foot, without any aid from the hand of the wearer, or other extrinſic application."

It is evident, therefore, that an artificial leg, poſſeſſing all the principal qualities aſcribed by Mr. P. to his invention, particularly the flexion of the knee-joint in walking, by which ſemicircular motion is avoided, and the ſelf-acting power by which it accommodates itſelf to almoſt any natural motion without any aſſiſtance from the hands, was ſeveral years ſince preſented to the public by Mr. Mann, of Bradford in Yorkſhire; and that his machine has been, and is conſidered worthy their attention, the following recommendation will teſtify.

"The public are hereby certified, that we think Mr. Mann's invention not only highly intereſting to all who have had the misfortune to loſe a leg, but worthy of the patronage of any profeſſional gentleman.

John Hunter, Surgeon General to his Majeſty's Forces.

T. Keate, Surgeon to his Majeſty's Royal Hoſpital, Chelſea.

John Gunning, Maſter

W. Grindall, Warden

W. Lucas, Warden

Jos. Warner, Aſſiſtant

Henry Wadron, Aſſiſtant

Edmund Pitts, Aſſiſtant

James Patch, Aſſiſtant

W. Cooper, Aſſiſtant

} of the Corporation of Surgeons, London.

G. Chandler,

G. Chandler, }

John Birch, } Surgeons to St. Thomas's Hofpital.

Henry Cline, }

W. Hey, Surgeon to the Infirmary, Leeds.

Charles White, F. R. S. Surgeon to the Infirmary, Manchefter."

On the comparative merits of the two inventions, experience will beft determine, and to that teft I wifh to commit their refpective claims; but in juftice to Mr. Mann, I think it fair to affert his title to the credit of having *first* difcovered, and introduced to public notice, an inftrument fo capable of alleviating one of the greateft calamities incident to humanity.

I am, yours, &c.

*April* 11, 1801. T. R. REID, Surgeon, Chelfea.

